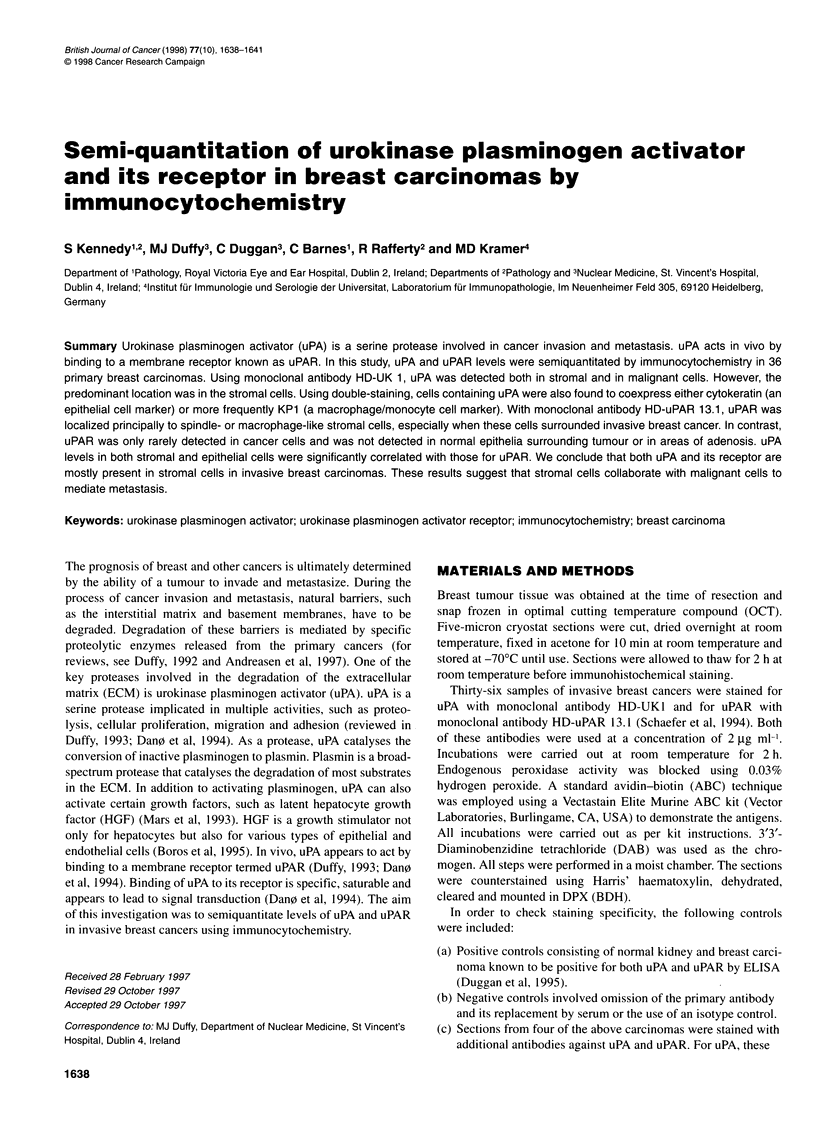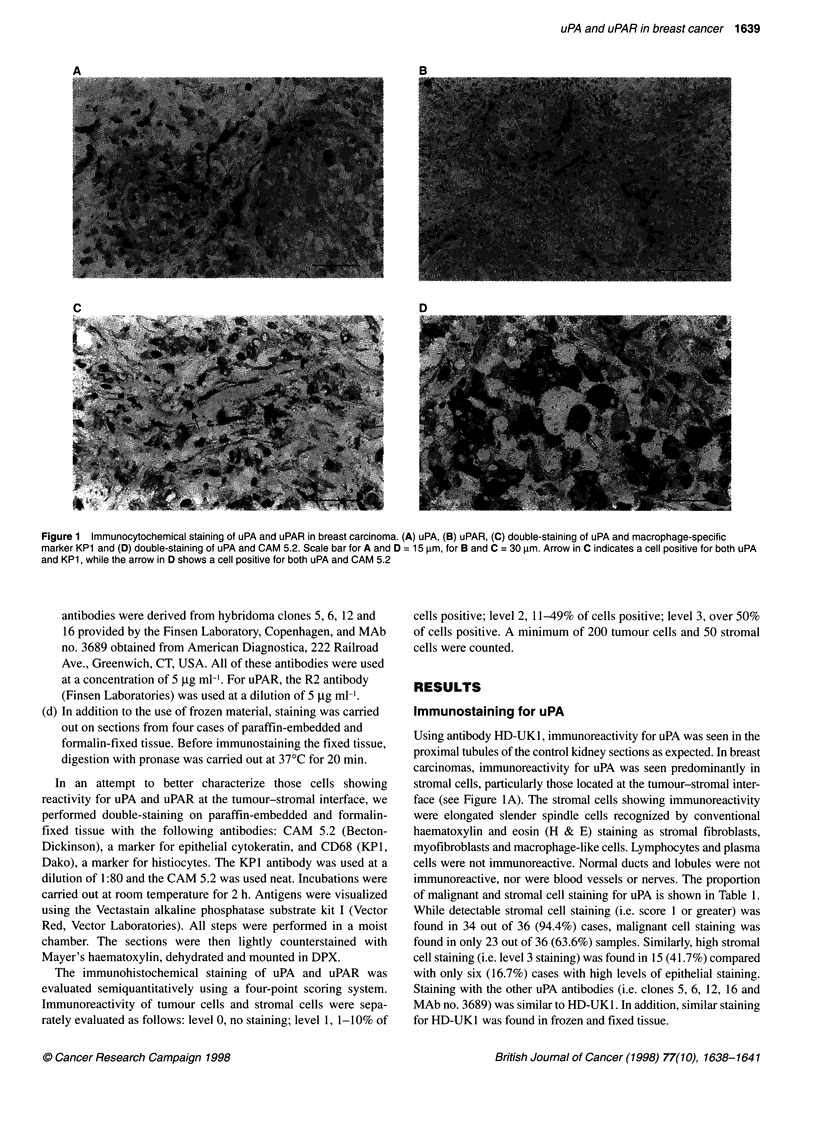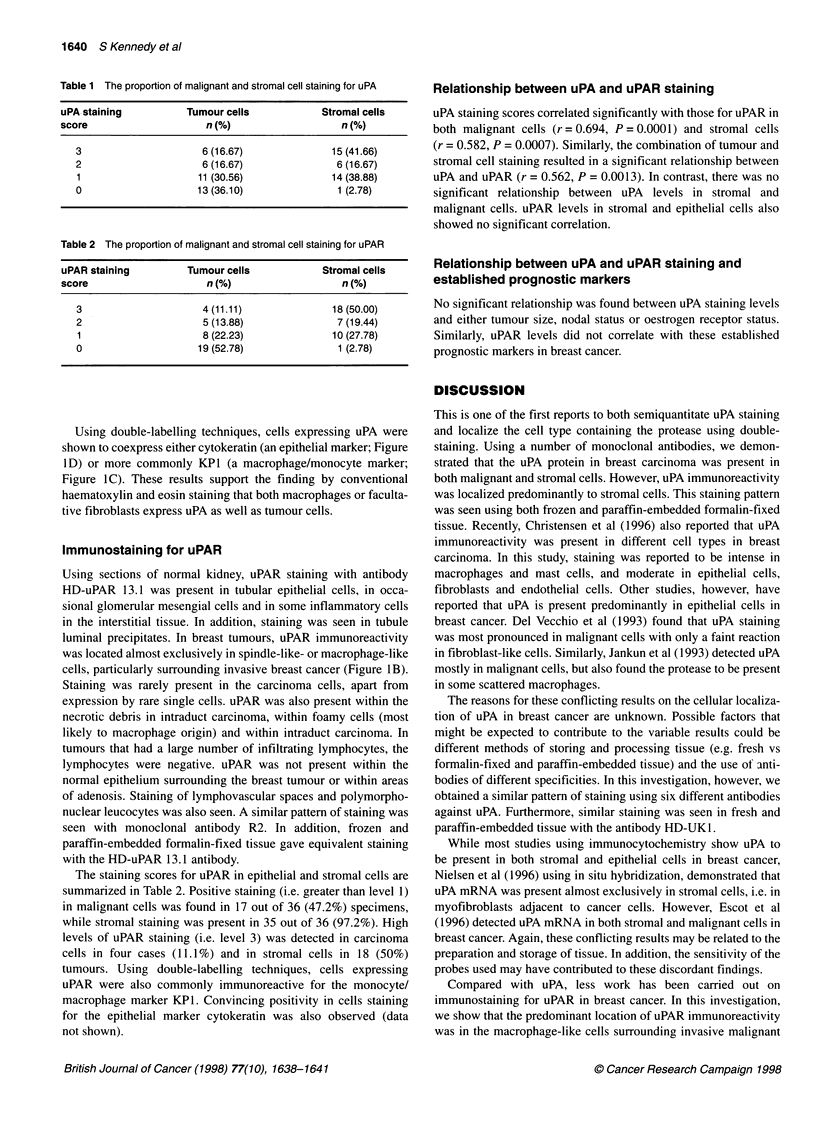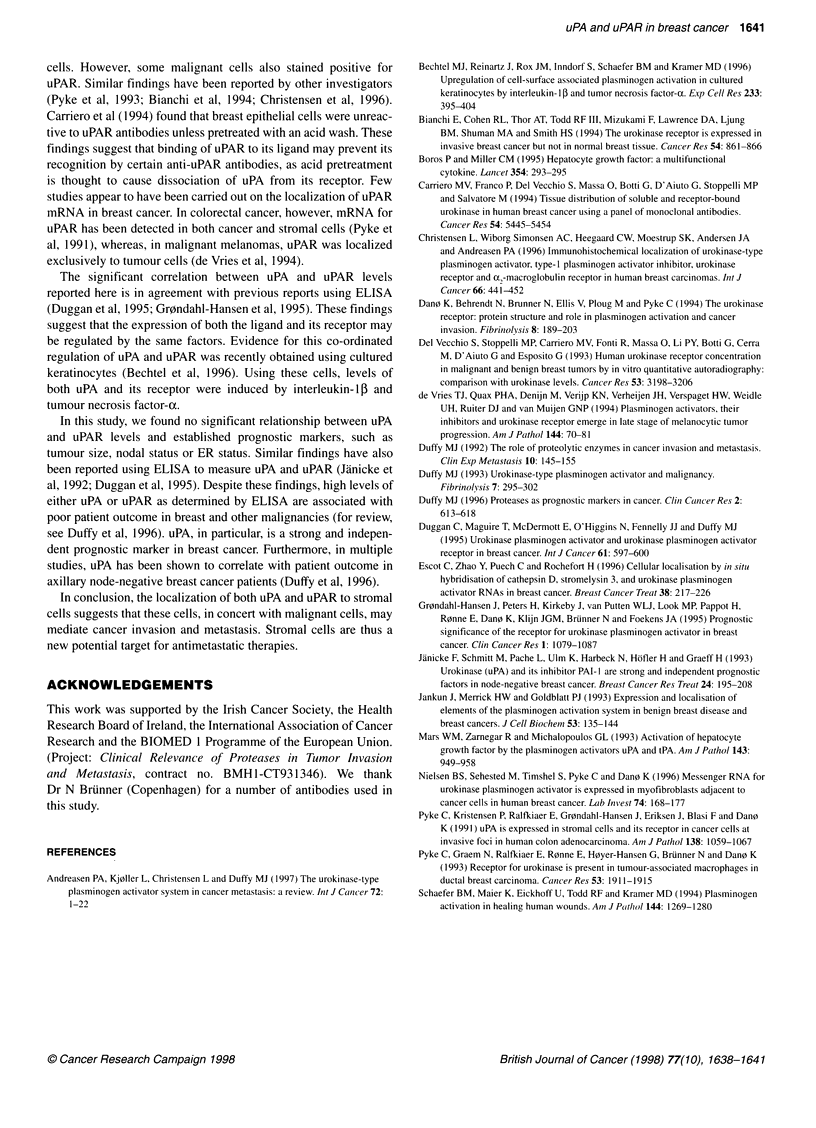# Semi-quantitation of urokinase plasminogen activator and its receptor in breast carcinomas by immunocytochemistry.

**DOI:** 10.1038/bjc.1998.268

**Published:** 1998-05

**Authors:** S. Kennedy, M. J. Duffy, C. Duggan, C. Barnes, R. Rafferty, M. D. Kramer

**Affiliations:** Department of Pathology, Royal Victoria Eye and Ear Hospital, Dublin, Ireland.

## Abstract

**Images:**


British Journal of Cancer (1998) 77(10), 1638–1641
© 1998 Cancer Research Campaign

### Semi-quantitation of urokinase plasminogen activator and its receptor in breast carcinomas by immunocytochemistry

**S Kennedy[1,2], MJ Duffy[3], C Duggan[3], C Barnes[1], R Rafferty[2] and MD Kramer[4]**

Department of [1]Pathology, Royal Victoria Eye and Ear Hospital, Dublin 2, Ireland; Departments of [2]Pathology and [3]Nuclear Medicine, St. Vincent's Hospital, Dublin 4, Ireland; [4]Institut für Immunologie und Serologie der Universitat, Laboratorium für Immunopathologie, Im Neuenheimer Feld 305, 69120 Heidelberg, Germany

**Summary** Urokinase plasminogen activator (uPA) is a serine protease involved in cancer invasion and metastasis. uPA acts in vivo by binding to a membrane receptor known as uPAR. In this study, uPA and uPAR levels were semiquantitated by immunocytochemistry in 36 primary breast carcinomas. Using monoclonal antibody HD-UK 1, uPA was detected both in stromal and in malignant cells. However, the predominant location was in the stromal cells. Using double-staining, cells containing uPA were also found to coexpress either cytokeratin (an epithelial cell marker) or more frequently KP1 (a macrophage/monocyte cell marker). With monoclonal antibody HD-uPAR 13.1, uPAR was localized principally to spindle- or macrophage-like stromal cells, especially when these cells surrounded invasive breast cancer. In contrast, uPAR was only rarely detected in cancer cells and was not detected in normal epithelia surrounding tumour or in areas of adenosis. uPA levels in both stromal and epithelial cells were significantly correlated with those for uPAR. We conclude that both uPA and its receptor are mostly present in stromal cells in invasive breast carcinomas. These results suggest that stromal cells collaborate with malignant cells to mediate metastasis.

**Keywords:** urokinase plasminogen activator; urokinase plasminogen activator receptor; immunocytochemistry; breast carcinoma

The prognosis of breast and other cancers is ultimately determined by the ability of a tumour to invade and metastasize. During the process of cancer invasion and metastasis, natural barriers, such as the interstitial matrix and basement membranes, have to be degraded. Degradation of these barriers is mediated by specific proteolytic enzymes released from the primary cancers (for reviews, see Duffy, 1992 and Andreasen et al, 1997). One of the key proteases involved in the degradation of the extracellular matrix (ECM) is urokinase plasminogen activator (uPA). uPA is a serine protease implicated in multiple activities, such as proteolysis, cellular proliferation, migration and adhesion (reviewed in Duffy, 1993; Danø et al, 1994). As a protease, uPA catalyses the conversion of inactive plasminogen to plasmin. Plasmin is a broad-spectrum protease that catalyses the degradation of most substrates in the ECM. In addition to activating plasminogen, uPA can also activate certain growth factors, such as latent hepatocyte growth factor (HGF) (Mars et al, 1993). HGF is a growth stimulator not only for hepatocytes but also for various types of epithelial and endothelial cells (Boros et al, 1995). In vivo, uPA appears to act by binding to a membrane receptor termed uPAR (Duffy, 1993; Danø et al, 1994). Binding of uPA to its receptor is specific, saturable and appears to lead to signal transduction (Danø et al, 1994). The aim of this investigation was to semiquantitate levels of uPA and uPAR in invasive breast cancers using immunocytochemistry.

*Received 28 February 1997*
*Revised 29 October 1997*
*Accepted 29 October 1997*

*Correspondence to:* MJ Duffy, Department of Nuclear Medicine, St Vincent's Hospital, Dublin 4, Ireland

### MATERIALS AND METHODS

Breast tumour tissue was obtained at the time of resection and snap frozen in optimal cutting temperature compound (OCT). Five-micron cryostat sections were cut, dried overnight at room temperature, fixed in acetone for 10 min at room temperature and stored at –70°C until use. Sections were allowed to thaw for 2 h at room temperature before immunohistochemical staining.

Thirty-six samples of invasive breast cancers were stained for uPA with monoclonal antibody HD-UK1 and for uPAR with monoclonal antibody HD-uPAR 13.1 (Schaefer et al, 1994). Both of these antibodies were used at a concentration of 2 $\mu g\ ml^{-1}$. Incubations were carried out at room temperature for 2 h. Endogenous peroxidase activity was blocked using 0.03% hydrogen peroxide. A standard avidin–biotin (ABC) technique was employed using a Vectastain Elite Murine ABC kit (Vector Laboratories, Burlingame, CA, USA) to demonstrate the antigens. All incubations were carried out as per kit instructions. 3′3′-Diaminobenzidine tetrachloride (DAB) was used as the chromogen. All steps were performed in a moist chamber. The sections were counterstained using Harris' haematoxylin, dehydrated, cleared and mounted in DPX (BDH).

In order to check staining specificity, the following controls were included:

(a) Positive controls consisting of normal kidney and breast carcinoma known to be positive for both uPA and uPAR by ELISA (Duggan et al, 1995).
(b) Negative controls involved omission of the primary antibody and its replacement by serum or the use of an isotype control.
(c) Sections from four of the above carcinomas were stained with additional antibodies against uPA and uPAR. For uPA, these

**Figure 1** Immunocytochemical staining of uPA and uPAR in breast carcinoma. (**A**) uPA, (**B**) uPAR, (**C**) double-staining of uPA and macrophage-specific marker KP1 and (**D**) double-staining of uPA and CAM 5.2. Scale bar for **A** and **D** = 15 μm, for **B** and **C** = 30 μm. Arrow in **C** indicates a cell positive for both uPA and KP1, while the arrow in **D** shows a cell positive for both uPA and CAM 5.2

antibodies were derived from hybridoma clones 5, 6, 12 and 16 provided by the Finsen Laboratory, Copenhagen, and MAb no. 3689 obtained from American Diagnostica, 222 Railroad Ave., Greenwich, CT, USA. All of these antibodies were used at a concentration of 5 μg $ml^{-1}$. For uPAR, the R2 antibody (Finsen Laboratories) was used at a dilution of 5 μg $ml^{-1}$.

(d) In addition to the use of frozen material, staining was carried out on sections from four cases of paraffin-embedded and formalin-fixed tissue. Before immunostaining the fixed tissue, digestion with pronase was carried out at 37°C for 20 min.

In an attempt to better characterize those cells showing reactivity for uPA and uPAR at the tumour–stromal interface, we performed double-staining on paraffin-embedded and formalin-fixed tissue with the following antibodies: CAM 5.2 (Becton-Dickinson), a marker for epithelial cytokeratin, and CD68 (KP1, Dako), a marker for histiocytes. The KP1 antibody was used at a dilution of 1:80 and the CAM 5.2 was used neat. Incubations were carried out at room temperature for 2 h. Antigens were visualized using the Vectastain alkaline phosphatase substrate kit I (Vector Red, Vector Laboratories). All steps were performed in a moist chamber. The sections were then lightly counterstained with Mayer's haematoxylin, dehydrated and mounted in DPX.

The immunohistochemical staining of uPA and uPAR was evaluated semiquantitatively using a four-point scoring system. Immunoreactivity of tumour cells and stromal cells were separately evaluated as follows: level 0, no staining; level 1, 1–10% of cells positive; level 2, 11–49% of cells positive; level 3, over 50% of cells positive. A minimum of 200 tumour cells and 50 stromal cells were counted.

### RESULTS

### Immunostaining for uPA

Using antibody HD-UK1, immunoreactivity for uPA was seen in the proximal tubules of the control kidney sections as expected. In breast carcinomas, immunoreactivity for uPA was seen predominantly in stromal cells, particularly those located at the tumour–stromal interface (see Figure 1A). The stromal cells showing immunoreactivity were elongated slender spindle cells recognized by conventional haematoxylin and eosin (H & E) staining as stromal fibroblasts, myofibroblasts and macrophage-like cells. Lymphocytes and plasma cells were not immunoreactive. Normal ducts and lobules were not immunoreactive, nor were blood vessels or nerves. The proportion of malignant and stromal cell staining for uPA is shown in Table 1. While detectable stromal cell staining (i.e. score 1 or greater) was found in 34 out of 36 (94.4%) cases, malignant cell staining was found in only 23 out of 36 (63.6%) samples. Similarly, high stromal cell staining (i.e. level 3 staining) was found in 15 (41.7%) compared with only six (16.7%) cases with high levels of epithelial staining. Staining with the other uPA antibodies (i.e. clones 5, 6, 12, 16 and MAb no. 3689) was similar to HD-UK1. In addition, similar staining for HD-UK1 was found in frozen and fixed tissue.

© Cancer Research Campaign 1998

Table 1 The proportion of malignant and stromal cell staining for uPA

| uPA staining score | Tumour cells n (%) | Stromal cells n (%) |
|---|---|---|
| 3 | 6 (16.67) | 15 (41.66) |
| 2 | 6 (16.67) | 6 (16.67) |
| 1 | 11 (30.56) | 14 (38.88) |
| 0 | 13 (36.10) | 1 (2.78) |

Table 2 The proportion of malignant and stromal cell staining for uPAR

| uPAR staining score | Tumour cells n (%) | Stromal cells n (%) |
|---|---|---|
| 3 | 4 (11.11) | 18 (50.00) |
| 2 | 5 (13.88) | 7 (19.44) |
| 1 | 8 (22.23) | 10 (27.78) |
| 0 | 19 (52.78) | 1 (2.78) |

Using double-labelling techniques, cells expressing uPA were shown to coexpress either cytokeratin (an epithelial marker; Figure 1D) or more commonly KP1 (a macrophage/monocyte marker; Figure 1C). These results support the finding by conventional haematoxylin and eosin staining that both macrophages or facultative fibroblasts express uPA as well as tumour cells.

### Immunostaining for uPAR

Using sections of normal kidney, uPAR staining with antibody HD-uPAR 13.1 was present in tubular epithelial cells, in occasional glomerular mesengial cells and in some inflammatory cells in the interstitial tissue. In addition, staining was seen in tubule luminal precipitates. In breast tumours, uPAR immunoreactivity was located almost exclusively in spindle-like- or macrophage-like cells, particularly surrounding invasive breast cancer (Figure 1B). Staining was rarely present in the carcinoma cells, apart from expression by rare single cells. uPAR was also present within the necrotic debris in intraduct carcinoma, within foamy cells (most likely to macrophage origin) and within intraduct carcinoma. In tumours that had a large number of infiltrating lymphocytes, the lymphocytes were negative. uPAR was not present within the normal epithelium surrounding the breast tumour or within areas of adenosis. Staining of lymphovascular spaces and polymorphonuclear leucocytes was also seen. A similar pattern of staining was seen with monoclonal antibody R2. In addition, frozen and paraffin-embedded formalin-fixed tissue gave equivalent staining with the HD-uPAR 13.1 antibody.

The staining scores for uPAR in epithelial and stromal cells are summarized in Table 2. Positive staining (i.e. greater than level 1) in malignant cells was found in 17 out of 36 (47.2%) specimens, while stromal staining was present in 35 out of 36 (97.2%). High levels of uPAR staining (i.e. level 3) was detected in carcinoma cells in four cases (11.1%) and in stromal cells in 18 (50%) tumours. Using double-labelling techniques, cells expressing uPAR were also commonly immunoreactive for the monocyte/macrophage marker KP1. Convincing positivity in cells staining for the epithelial marker cytokeratin was also observed (data not shown).

### Relationship between uPA and uPAR staining uPA staining scores correlated significantly with those for uPAR in both malignant cells ($r = 0.694$, $P = 0.0001$) and stromal cells ($r = 0.582$, $P = 0.0007$). Similarly, the combination of tumour and stromal cell staining resulted in a significant relationship between uPA and uPAR ($r = 0.562$, $P = 0.0013$). In contrast, there was no significant relationship between uPA levels in stromal and malignant cells. uPAR levels in stromal and epithelial cells also showed no significant correlation.

### Relationship between uPA and uPAR staining and established prognostic markers

No significant relationship was found between uPA staining levels and either tumour size, nodal status or oestrogen receptor status. Similarly, uPAR levels did not correlate with these established prognostic markers in breast cancer.

### DISCUSSION

This is one of the first reports to both semiquantitate uPA staining and localize the cell type containing the protease using double-staining. Using a number of monoclonal antibodies, we demonstrated that the uPA protein in breast carcinoma was present in both malignant and stromal cells. However, uPA immunoreactivity was localized predominantly to stromal cells. This staining pattern was seen using both frozen and paraffin-embedded formalin-fixed tissue. Recently, Christensen et al (1996) also reported that uPA immunoreactivity was present in different cell types in breast carcinoma. In this study, staining was reported to be intense in macrophages and mast cells, and moderate in epithelial cells, fibroblasts and endothelial cells. Other studies, however, have reported that uPA is present predominantly in epithelial cells in breast cancer. Del Vecchio et al (1993) found that uPA staining was most pronounced in malignant cells with only a faint reaction in fibroblast-like cells. Similarly, Jankun et al (1993) detected uPA mostly in malignant cells, but also found the protease to be present in some scattered macrophages.

The reasons for these conflicting results on the cellular localization of uPA in breast cancer are unknown. Possible factors that might be expected to contribute to the variable results could be different methods of storing and processing tissue (e.g. fresh vs formalin-fixed and paraffin-embedded tissue) and the use of antibodies of different specificities. In this investigation, however, we obtained a similar pattern of staining using six different antibodies against uPA. Furthermore, similar staining was seen in fresh and paraffin-embedded tissue with the antibody HD-UK1.

While most studies using immunocytochemistry show uPA to be present in both stromal and epithelial cells in breast cancer, Nielsen et al (1996) using in situ hybridization, demonstrated that uPA mRNA was present almost exclusively in stromal cells, i.e. in myofibroblasts adjacent to cancer cells. However, Escot et al (1996) detected uPA mRNA in both stromal and malignant cells in breast cancer. Again, these conflicting results may be related to the preparation and storage of tissue. In addition, the sensitivity of the probes used may have contributed to these discordant findings.

Compared with uPA, less work has been carried out on immunostaining for uPAR in breast cancer. In this investigation, we show that the predominant location of uPAR immunoreactivity was in the macrophage-like cells surrounding invasive malignant

© Cancer Research Campaign 1998

cells. However, some malignant cells also stained positive for uPAR. Similar findings have been reported by other investigators (Pyke et al, 1993; Bianchi et al, 1994; Christensen et al, 1996). Carriero et al (1994) found that breast epithelial cells were unreactive to uPAR antibodies unless pretreated with an acid wash. These findings suggest that binding of uPAR to its ligand may prevent its recognition by certain anti-uPAR antibodies, as acid pretreatment is thought to cause dissociation of uPA from its receptor. Few studies appear to have been carried out on the localization of uPAR mRNA in breast cancer. In colorectal cancer, however, mRNA for uPAR has been detected in both cancer and stromal cells (Pyke et al, 1991), whereas, in malignant melanomas, uPAR was localized exclusively to tumour cells (de Vries et al, 1994).

The significant correlation between uPA and uPAR levels reported here is in agreement with previous reports using ELISA (Duggan et al, 1995; Grøndahl-Hansen et al, 1995). These findings suggest that the expression of both the ligand and its receptor may be regulated by the same factors. Evidence for this co-ordinated regulation of uPA and uPAR was recently obtained using cultured keratinocytes (Bechtel et al, 1996). Using these cells, levels of both uPA and its receptor were induced by interleukin-1β and tumour necrosis factor-α.

In this study, we found no significant relationship between uPA and uPAR levels and established prognostic markers, such as tumour size, nodal status or ER status. Similar findings have also been reported using ELISA to measure uPA and uPAR (Jänicke et al, 1992; Duggan et al, 1995). Despite these findings, high levels of either uPA or uPAR as determined by ELISA are associated with poor patient outcome in breast and other malignancies (for review, see Duffy et al, 1996). uPA, in particular, is a strong and independent prognostic marker in breast cancer. Furthermore, in multiple studies, uPA has been shown to correlate with patient outcome in axillary node-negative breast cancer patients (Duffy et al, 1996).

In conclusion, the localization of both uPA and uPAR to stromal cells suggests that these cells, in concert with malignant cells, may mediate cancer invasion and metastasis. Stromal cells are thus a new potential target for antimetastatic therapies.

### ACKNOWLEDGEMENTS

This work was supported by the Irish Cancer Society, the Health Research Board of Ireland, the International Association of Cancer Research and the BIOMED 1 Programme of the European Union. (Project: *Clinical Relevance of Proteases in Tumor Invasion and Metastasis*, contract no. BMH1-CT931346). We thank Dr N Brünner (Copenhagen) for a number of antibodies used in this study.

### REFERENCES

Andreasen PA, Kjøller L, Christensen L and Duffy MJ (1997) The urokinase-type plasminogen activator system in cancer metastasis: a review. *Int J Cancer* **72**: 1–22

Bechtel MJ, Reinartz J, Rox JM, Inndorf S, Schaefer BM and Kramer MD (1996) Upregulation of cell-surface associated plasminogen activation in cultured keratinocytes by interleukin-1β and tumor necrosis factor-α. *Exp Cell Res* **233**: 395–404

Bianchi E, Cohen RL, Thor AT, Todd RF III, Mizukami F, Lawrence DA, Ljung BM, Shuman MA and Smith HS (1994) The urokinase receptor is expressed in invasive breast cancer but not in normal breast tissue. *Cancer Res* **54**: 861–866

Boros P and Miller CM (1995) Hepatocyte growth factor: a multifunctional cytokine. *Lancet* **354**: 293–295

Carriero MV, Franco P, Del Vecchio S, Massa O, Botti G, D'Aiuto G, Stoppelli MP and Salvatore M (1994) Tissue distribution of soluble and receptor-bound urokinase in human breast cancer using a panel of monoclonal antibodies. *Cancer Res* **54**: 5445–5454

Christensen L, Wiborg Simonsen AC, Heegaard CW, Moestrup SK, Andersen JA and Andreasen PA (1996) Immunohistochemical localization of urokinase-type plasminogen activator, type-1 plasminogen activator inhibitor, urokinase receptor and $\alpha_2$-macroglobulin receptor in human breast carcinomas. *Int J Cancer* **66**: 441–452

Danø K, Behrendt N, Brunner N, Ellis V, Ploug M and Pyke C (1994) The urokinase receptor: protein structure and role in plasminogen activation and cancer invasion. *Fibrinolysis* **8**: 189–203

Del Vecchio S, Stoppelli MP, Carriero MV, Fonti R, Massa O, Li PY, Botti G, Cerra M, D'Aiuto G and Esposito G (1993) Human urokinase receptor concentration in malignant and benign breast tumors by in vitro quantitative autoradiography: comparison with urokinase levels. *Cancer Res* **53**: 3198–3206

de Vries TJ, Quax PHA, Denijn M, Verijp KN, Verheijen JH, Verspaget HW, Weidle UH, Ruiter DJ and van Muijen GNP (1994) Plasminogen activators, their inhibitors and urokinase receptor emerge in late stage of melanocytic tumor progression. *Am J Pathol* **144**: 70–81

Duffy MJ (1992) The role of proteolytic enzymes in cancer invasion and metastasis. *Clin Exp Metastasis* **10**: 145–155

Duffy MJ (1993) Urokinase-type plasminogen activator and malignancy. *Fibrinolysis* **7**: 295–302

Duffy MJ (1996) Proteases as prognostic markers in cancer. *Clin Cancer Res* **2**: 613–618

Duggan C, Maguire T, McDermott E, O'Higgins N, Fennelly JJ and Duffy MJ (1995) Urokinase plasminogen activator and urokinase plasminogen activator receptor in breast cancer. *Int J Cancer* **61**: 597–600

Escot C, Zhao Y, Puech C and Rochefort H (1996) Cellular localisation by *in situ* hybridisation of cathepsin D, stromelysin 3, and urokinase plasminogen activator RNAs in breast cancer. *Breast Cancer Treat* **38**: 217–226

Grøndahl-Hansen J, Peters H, Kirkeby J, van Putten WLJ, Look MP, Pappot H, Rønne E, Danø K, Klijn JGM, Brünner N and Foekens JA (1995) Prognostic significance of the receptor for urokinase plasminogen activator in breast cancer. *Clin Cancer Res* **1**: 1079–1087

Jänicke F, Schmitt M, Pache L, Ulm K, Harbeck N, Höfler H and Graeff H (1993) Urokinase (uPA) and its inhibitor PAI-1 are strong and independent prognostic factors in node-negative breast cancer. *Breast Cancer Res Treat* **24**: 195–208

Jankun J, Merrick HW and Goldblatt PJ (1993) Expression and localisation of elements of the plasminogen activation system in benign breast disease and breast cancers. *J Cell Biochem* **53**: 135–144

Mars WM, Zarnegar R and Michalopoulos GL (1993) Activation of hepatocyte growth factor by the plasminogen activators uPA and tPA. *Am J Pathol* **143**: 949–958

Nielsen BS, Sehested M, Timshel S, Pyke C and Danø K (1996) Messenger RNA for urokinase plasminogen activator is expressed in myofibroblasts adjacent to cancer cells in human breast cancer. *Lab Invest* **74**: 168–177

Pyke C, Kristensen P, Ralfkiaer E, Grøndahl-Hansen J, Eriksen J, Blasi F and Danø K (1991) uPA is expressed in stromal cells and its receptor in cancer cells at invasive foci in human colon adenocarcinoma. *Am J Pathol* **138**: 1059–1067

Pyke C, Graem N, Ralfkiaer E, Rønne E, Høyer-Hansen G, Brünner N and Danø K (1993) Receptor for urokinase is present in tumour-associated macrophages in ductal breast carcinoma. *Cancer Res* **53**: 1911–1915

Schaefer BM, Maier K, Eickhoff U, Todd RF and Kramer MD (1994) Plasminogen activation in healing human wounds. *Am J Pathol* **144**: 1269–1280

© Cancer Research Campaign 1998